# Development and validation of a programmed cell death index to predict the prognosis and drug sensitivity of gastric cancer

**DOI:** 10.3389/fphar.2024.1477363

**Published:** 2024-12-18

**Authors:** Feizhi Lin, Xiaojiang Chen, Chengcai Liang, Ruopeng Zhang, Guoming Chen, Ziqi Zheng, Bowen Huang, Chengzhi Wei, Zhoukai Zhao, Feiyang Zhang, Zewei Chen, Shenghang Ruan, Yongming Chen, Runcong Nie, Yuangfang Li, Baiwei Zhao

**Affiliations:** State Key Laboratory of Oncology in South China, Department of Gastric Surgery, Sun Yat-Sen University Cancer Center, Guangdong Provincial Clinical Research Center for Cancer, Guangzhou, China

**Keywords:** gastric cancer, programmed cell death, prognostic model, drug sensitivity, tumor microenvironment

## Abstract

**Aim:**

Programmed cell death (PCD) critically influences the tumor microenvironment (TME) and is intricately linked to tumor progression and patient prognosis. This study aimed to develop a novel prognostic indicator and marker of drug sensitivity in patients with gastric cancer (GC) based on PCD.

**Methods:**

We analyzed genes associated with 14 distinct PCD patterns using bulk transcriptome data and clinical information from TCGA-STAD for model construction with univariate Cox regression and LASSO regression analyses. Microarray data from GSE62254, GSE15459, and GSE26901 were used for validation. Single-cell transcriptome data from GSE183904 were analyzed to explore the relationship between TME and the newly constructed model, named PCD index (PCDI). Drug sensitivity comparisons were made between patients with high and low PCDI scores.

**Results:**

We developed a novel twelve-gene signature called PCDI. Upon validation, GC patients with higher PCDI scores had poorer prognoses. A high-performance nomogram integrating the PCDI with clinical features was also established. Additionally, single-cell transcriptome data analysis suggested that PCDI might be linked to critical components of the TME. Patients with high PCDI scores exhibited resistance to standard adjuvant chemotherapy and immunotherapy but might benefit from targeted treatments with NU7441, Dasatinib, and JQ1.

**Conclusion:**

The novel PCDI model shows significant potential in predicting clinical prognosis and drug sensitivity of GC, thereby facilitating personalized treatment strategies for patients with GC.

## 1 Introduction

Gastric cancer (GC) is a major contributor to the global cancer burden, with over 968,000 new cases and nearly 660,000 deaths reported in 2022, ranking fifth in terms of both incidence and mortality worldwide (Bray et al., 2024). Patients are often diagnosed at advanced stages owing to subtle early symptoms, and the prognosis for patients with advanced GC remains unsatisfactory despite advancements in treatments such as improved surgical techniques, optimized chemotherapy and the introduction of immunotherapy and targeted therapy (Guan et al., 2023). The American Joint Committee on Cancer (AJCC) TNM staging system is widely used to stage for GC. Recently, the molecular classification of GC has gradually attracted people’s attention. The molecular subtypes of The Cancer Genome Atlas (TCGA) include EBV (Epstein-Barr virus), MSI (microsatellite instability), CIN (chromosomal instability), and GS (genomically stable), while those of the Asian Cancer Research Group (ACRG) include MSI-H (microsatellite instability-high), MSS (microsatellite stable)/TP53+, MSS/TP53-, and MSS/EMT (epithelial-mesenchymal transition) (Cancer Genome Atlas Research Network, 2014; Cristescu et al., 2015). These classifications facilitate stratification of treatment approaches for GC. However, the variability in drug response and clinical outcomes, even within the same AJCC TNM stage or molecular subtype, highlights the need for a refined classification system to personalize diagnostic and therapeutic strategies for patients with GC.

Programmed cell death (PCD) is a kind of genetic control, autonomous and orderly important cell death that involves the activation, expression, and regulation of a series of genes and encompasses multiple patterns (Chen et al., 2024). Apoptosis is characterized by caspase-mediated pathways that lead to cellular shrinkage, DNA fragmentation, and the formation of apoptotic bodies, facilitating controlled cell elimination without provoking inflammation (Obeng, 2021). Necroptosis promotes inflammation via the necrotic death pathway, which serves as an alternative to apoptosis when caspase activation is inhibited (Vandenabeele et al., 2010). Autophagy degrades cellular components under stress and is linked to pathologies, such as cancer and neurodegenerative diseases (Levine and Kroemer, 2008). Anoikis is a type of apoptosis triggered by the loss of cell attachment and prevents metastasis by inhibiting anchorage-independent growth, which is crucial for cancer progression (Simpson et al., 2008). Pyroptosis involves gasdermin-induced membrane pore formation, leading to cell lysis and inflammation (Zhou et al., 2022). Entotic cell death occurs when one cell engulfs another, potentially leading to the death of the internalized cell, which is indicative of the high cellular stress and observed in tumor environments (Tang et al., 2019). Parthanatos results from excessive PARP activation, which causes mitochondrial dysfunction and large-scale DNA fragmentation (Tang et al., 2019). Ferroptosis is driven by iron-dependent lipid peroxidation, which has been implicated in cancer and ischemic damage (Dixon et al., 2012). Netotic cell death involves the release of neutrophil extracellular traps (NETs), which help combat infections, but can also exacerbate chronic inflammation and thrombosis (Boeltz et al., 2019). Lysosome-dependent cell death occurs via lysosomal membrane rupture, releasing cathepsins that catalyze apoptosis or necrosis, which are relevant in cancer therapy (Boya and Kroemer, 2008). Oxeiptosis is caspase-independent and triggered by oxidative stress through the KEAP1-PGAM5-AIFM1 axis (Holze et al., 2018). Disulfidptosis involves the disruption of protein disulfide bonds, which are critical for maintaining cellular function and integrity, particularly under oxidative stress conditions (Liu et al., 2024). Cuproptosis results from Cu accumulation, which causes lipid oxidation and mitochondrial dysfunction (Xie et al., 2023). Alkaliptosis is characterized by an increase in intracellular pH, leading to the dysregulation of metabolic and ion transport (Liu et al., 2020). Overall, PCD involves diverse mechanisms and plays a regulatory role in maintaining cellular and tissue homeostasis (Yuan and Ofengeim, 2024). The loss of control over single or mixed types of PCD can lead to human diseases, including various cancers (Chen et al., 2024). However, the comprehensive association between these 14 forms of PCD and GC remains unclear.

In this study, we aimed to identify PCD-related genes that significantly affect the prognosis of patients with GC and to develop a novel indicator, namely, programmed cell death index (PCDI) to predict their survival and therapeutic response. Additionally, we explored the potential relationship between the PCDI and the tumor microenvironment (TME) of GC.

## 2 Materials and methods

### 2.1 Data acquisition

Normalized and log2-transformed bulk RNA-sequencing (RNA-seq) profiles, expressed as transcripts per million (TPM), along with clinical and survival data for patients with GC and healthy individuals, were obtained from the University of California Santa Cruz (UCSC) database (https://xenabrowser.net/). The “gencode annotation v23”file was utilized for ID-gene mapping. Masked somatic mutation data were acquired using the “TCGAbiolinks” package (Colaprico et al., 2016). We also retrieved additional bulk RNA-seq and survival data for the validation cohorts (GSE62254, GSE15459, and GSE26901) and a single-cell RNA-seq profile from GSE183904 from the Gene Expression Omnibus (GEO) database (Clough and Barrett, 2016). Samples that lacked clinical information or exhibited more than 90% of the genes with zero expression were excluded. PCD-related genes were compiled from peer-reviewed publications, gene set enrichment analysis (GSEA), the Kyoto Encyclopedia of Genes and Genomes (KEGG), and the Gene Ontology (GO) databases (Zou et al., 2022; Subramanian et al., 2005; Kanehisa and Goto, 2000; Gene Ontology Consortium, 2015).

### 2.2 Identification of expression and variation levels of PCD-related genes

Differential expression analysis of GC and normal gastric tissue samples from the TCGA-STAD cohort was performed using the “limma” package, defining differential expressed genes (DEGs) with adjusted *p* < 0.05 and |log2FC| > 1 (Ritchie et al., 2015). Somatic mutation analysis was conducted using the “maftools” package (Mayakonda et al., 2018).

### 2.3 Prognostic gene signature development for GC patients

Significant survival-related genes were identified using univariate Cox regression analysis (*p* < 0.05). LASSO (least absolute shrinkage and selection operator) regression, implemented through the “glmnet” package, was further employed to refine variables, thereby avoiding collinearity and overfitting (Engebretsen and Bohlin, 2019). The “lambda.min” value was selected to control model complexity. The programmed cell death index (PCDI) for each patient was calculated as follows: PCDI = $\sum_{k=1}^{n}\text{Coef}k*\;\mathrm{Exp}k$ . Coef*k* represents the risk coefficient and Exp*k* represents the gene expression level. Patients were stratified into high and low PCDI groups based on the median PCDI. Principal component analysis (PCA) was conducted using the “stats” package, and Kaplan-Meier analysis was performed using the “survival” and “survminer” packages to explore the correlation between overall survival (OS) time and PCDI.

### 2.4 Unsupervised clustering of PCDI model genes

The “ConsensusClusterPlus” package was used for unsupervised clustering based on 12 model genes to identify GC subtypes (Wilkerson and Hayes, 2010). The consensus cluster parameters were set as pItem = 0.8, maxK = 9, clusterAlg = “km,” and distance = “pearson.” Kaplan-Meier analysis was used to compare the OS of patients with GC across different clusters.

### 2.5 Construction and validation of nomogram

Both univariate and multivariate Cox regression analyses were conducted to validate the PCDI as an independent prognostic indicator for patients with GC, assessing its significance along with relevant clinical parameters. A prognostic nomogram was developed for the TCGA-STAD cohort using the “rms” and “replot” packages. The nomogram’s performance was evaluated through calibration curves (“rms” package), decision curve analysis (DCA, “rmda” package), and receiver operating characteristic (ROC, “timeROC” package) curves (Blanche et al., 2013).

### 2.6 Immune cell infiltration and single-cell analysis

The “CIBERSORT” package was used to estimate the fractions of 22 types of immune cells in each sample, and the immune cell infiltration levels were compared between the low- and high-PCDI groups (Chen et al., 2018). Single-cell RNA-seq data analysis of GSE183904 was performed using the “Seurat” packages (Butler et al., 2018).

### 2.7 Functional enrichment analysis

Functional enrichment analyses were carried out using the “clusterProfiler” and “org.Hs.eg.db” packages to identify relevant KEGG pathways based on DEGs (Wu et al., 2021). Gene set variation analysis (GSVA) was utilized to analyze differences in KEGG pathways between the high- and low-PCDI groups using the “msigdbr,” “GSVA,” and “GSEABase” R packages (Liberzon et al., 2015; Hänzelmann et al., 2013). The possible GO biological processes of the marker genes in each cell type were also identified.

### 2.8 Drug sensitivity analysis

Drug sensitivities were predicted using the “oncoPredict” package, and the Tumor Immune Dysfunction and Exclusion (TIDE) algorithm (http://tide.dfci.harvard.edu/) was used to predict responses to immunotherapy between the low- and high-PCDI groups (Maeser et al., 2021).

### 2.9 Statistical analysis

All statistical analyses were conducted using the R software (version 4.2.3). For parametric variables, comparisons between different groups were made using Student’s t-test or one-way ANOVA. For nonparametric variables, the Wilcoxon rank-sum test or Kruskal–Wallis test was used. Correlations between variables were assessed using Spearman’s rank-order correlation for nonparametric data and Pearson’s r correlation for parametric data. Categorical data were analyzed using the Chi-square test. Survival analyses were performed using the log-rank test. Statistical significance was set at *p* < 0.05.

## 3 Results

### 3.1 Study workflow

**FIGURE 1:**
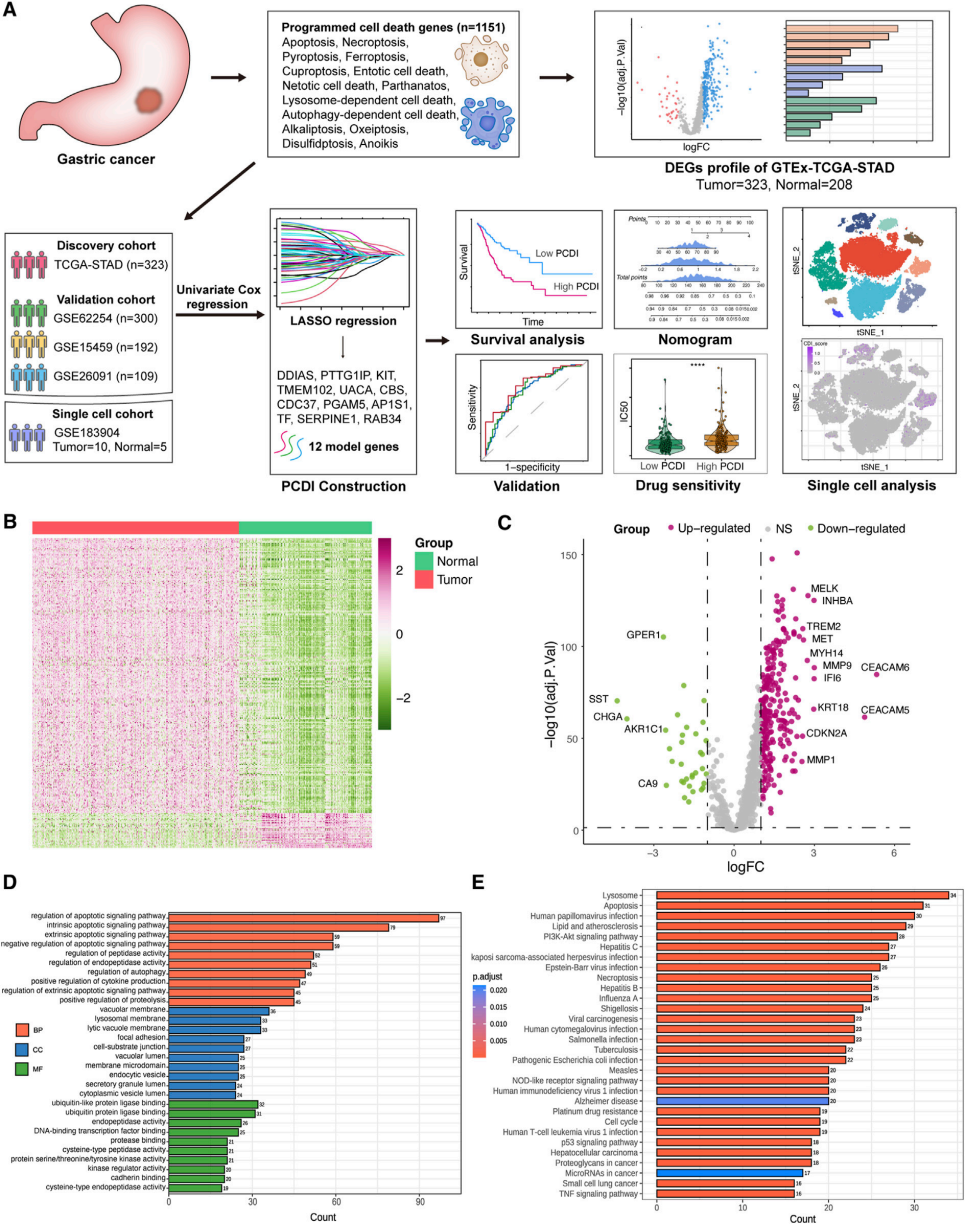
Workflow and DEGs landscape of PCD-Related Genes **(A)** Schematic diagram of the study’s design. **(B)** Heatmap displaying the PCD-related DEGs between GC and normal tissues in the TCGA-STAD cohort. **(C)** Volcano plot illustrating the PCD-related DEGs. Downregulated DEGs are indicated in green, upregulated DEGs in pink, and non-significant genes in grey. Labeled points represent DEGs with an adjusted *p*-value <0.01 and |log2FC| > 2.5. **(D)** GO enrichment analysis for the PCD-related DEGs. **(E)** KEGG enrichment analysis for the PCD-related DEGs. GC, gastric cancer. LASSO, least absolute shrinkage and selection operator. PCD, programmed cell death. DEGs, differentially expressed genes. logFC, log_2_ (fold change). GO, Gene Ontology. BP, biological process. CC, cellular component. MF, molecular function. KEGG, Kyoto Encyclopedia of Genes and Genomes.

After excluding samples that lacked clinical information or exhibited more than 90% of the genes with zero expression, we selected 323 patients with GC from the UCSC Xena database for the training cohort, along with 208 normal samples. Moreover, 300 patients from GSE62254, 192 from GSE15459, and 109 from GSE26901 were included in the GEO database as validation cohorts. We also incorporated single-cell RNA transcriptome datasets, including 10 GC samples and 5 normal samples from GSE183904. Moreover, after eliminating duplicate genes, a total of 1151 PCD-associated genes were included in subsequent analyses, including 580 apoptosis genes, 52 pyroptosis genes, 87 ferroptosis genes, 367 autophagy genes, 15 entotic cell death genes, 101 necroptosis genes, 14 cuproptosis genes, 9 parthanatos genes, 8 netotic cell death genes, 7 alkaliptosis genes, 220 lysosome-dependent cell death genes, 5 oxeiptosis genes, 17 disulfidptosis genes, and 36 anoikis genes (Supplementary Table 1). A flowchart of the study is shown in Figure 1A.

### 3.2 Landscape of PCD-related DEGs in GC

A total of 303 DEGs were identified in the TCGA-STAD cohort, with 269 upregulated and 34 downregulated genes in tumor samples compared to those in normal samples (adjusted *p* < 0.05, |log2FC| > 1). All DEGs are listed in Supplementary Table 2. Visualization of the scaled RNA levels of these DEGs is provided as heat maps in Figure 1B, and a volcano plot of these genes is shown in Figure 1C, with notable genes highlighted (|log2FC| > 2.5, adjusted *p* < 0.01). GO and KEGG pathway enrichment analyses revealed the involvement of these DEGs in pathways associated with apoptosis, autophagy, and necroptosis (Figures 1D, E). Mutation analysis of PCD related genes in patients with GC from the TCGA-STAD cohort showed that approximately 81.35% (253/311) had mutations, with TP53 having the highest mutation frequency at 46%, followed by others ranging from 7% to 16% (Supplementary Figure 1A).

### 3.3 Prognostic gene signature development for GC

**FIGURE 2:**
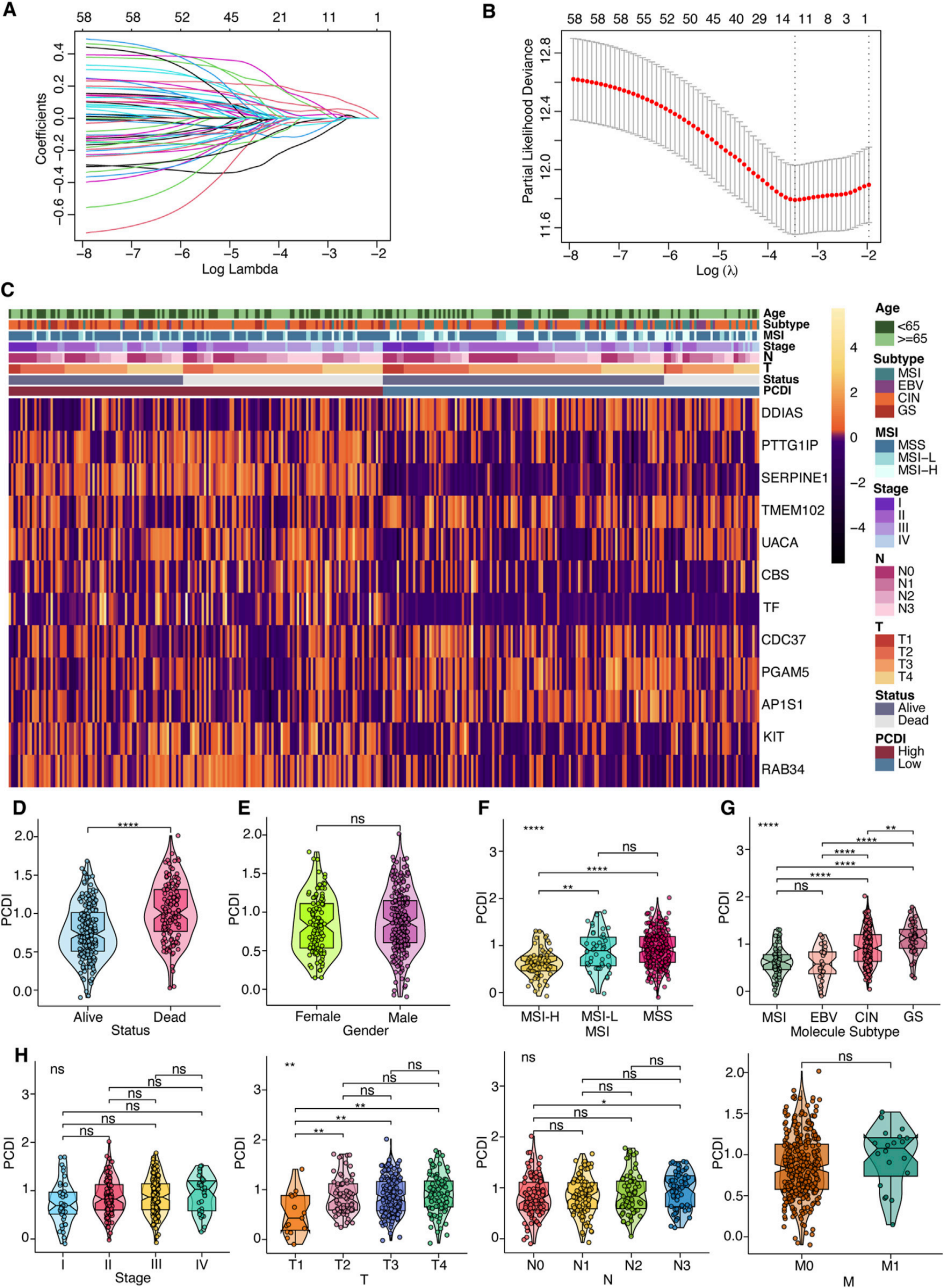
Prognostic Gene Signature Development for GC Patients **(A)** Selection of model genes by LASSO regression in the TCGA-STAD cohort. **(B)** Ten-fold cross-validation of the model. **(C)** Heatmap depicting the 12 model genes alongside clinical features. **(D–H)** Violin plots comparing differences in survival status, gender, MSI, TCGA molecular subtyping, stage, and TNM classification between high- and low-PCDI groups. Notations for significance are as follows: ns (not significant): *p* > 0.05; *: *p* ≤ 0.05; **: *p* ≤ 0.01; ***: *p* ≤ 0.001; ****: *p* ≤ 0.0001. PCDI, programmed cell death index. MSI, microsatellite instability. EBV, Epstein-Barr virus. CIN, chromosomal instability. GS, genomically stable. MSS, microsatellite stable. MSI-L, microsatellite instability-low. MSI-H, microsatellite instability-high. LASSO, least absolute shrinkage and selection operator.

Survival data were analyzed using univariate Cox regression, identifying 95 survival-related genes in the TCGA-STAD and 387 genes in the GSE62254 cohort, with a cutoff of *p* < 0.05. The intersection of these datasets yielded 60 genes, from which a 12-gene signature was developed using LASSO regression analysis in the TCGA-STAD cohort (Figures 2A, B). The genes comprising this signature included five from apoptosis, three from lysosome-dependent cell death, two from ferroptosis, one from autophagy, one from necroptosis, and one from oxeiptosis. The correlation between these genes is displayed in Supplementary Figure 2A. Expression levels were compared using the Wilcoxon test, and their influence on OS was assessed using Kaplan-Meier analysis (Supplementary Figures 2B, 3). We derived a programmed cell death index (PCDI) for each patient using the formula: PCDI = (−0.148926368 × DDIAS exp.) + (0.123056640 × PTTG1IP exp.) + (0.162336324 × SERPINE1 exp.) + (−0.025673067 × TMEM102 exp.) + (0.092603159 × UACA exp.) + (0.004036669 × CBS exp.) + (0.078952093 × TF exp.) + (−0.104077494 × CDC37 exp.) + (−0.042814168 × PGAM5 exp.) + (−0.019573107 × AP1S1 exp.) + (0.016993747 × KIT exp.) + (0.045710490 × RAB34 exp.). Patients in the TCGA-STAD cohort were stratified into high- and low-PCDI groups based on the median PCDI. We found that the PCDI was independent of the node stage, metastasis stage, and TNM stage. However, the PCDI was significantly associated with T stages (T1-T4), the survival status (alive or dead), MSI status, and GC TCGA molecular subtypes (Figures 2C–H). Notably, patients with T2-T4 stages and proficient mismatch repair (pMMR), defined as microsatellite stability (MSS) and low microsatellite instability (MSI-L), exhibited higher PCDI scores than those with high microsatellite instability (MSI-H). In terms of the TCGA molecular subtype, PCDI levels were ranked as follows: GS > CIN > EBV and MSI. Additionally, we investigated the mutation status of the top ten mutated PCD-related genes and found that mutations were more prevalent in the low-PCDI group for certain genes, such as PIK3CA, DIDO1, ATM, WDFY3, HERC1, BIRC6, and PRKDC (all *p* < 0.05; Supplementary Figure 1B). Among the model genes, AP1S1 exhibited more mutations in low-PCDI patients (*p* < 0.05; Supplementary Figure 1C). GSVA was employed to further explore the differences in the KEGG pathways among the two groups. Bar plots depicting the top ten upregulated and downregulated pathways in each cohort are presented in Supplementary Figures 4A–D. The most frequently upregulated pathways included “ECM receptor interaction” and “tryptophan metabolism,” while the most commonly downregulated pathway was “terpenoid backbone biosynthesis” (Supplementary Figure 4E). Additionally, nine PCD genes associated with these pathways were analyzed, and the protein-protein interaction (PPI) network analysis is displayed in Supplementary Figure 4F.

### 3.4 Training and validation of the gene signature prediction model

**FIGURE 3:**
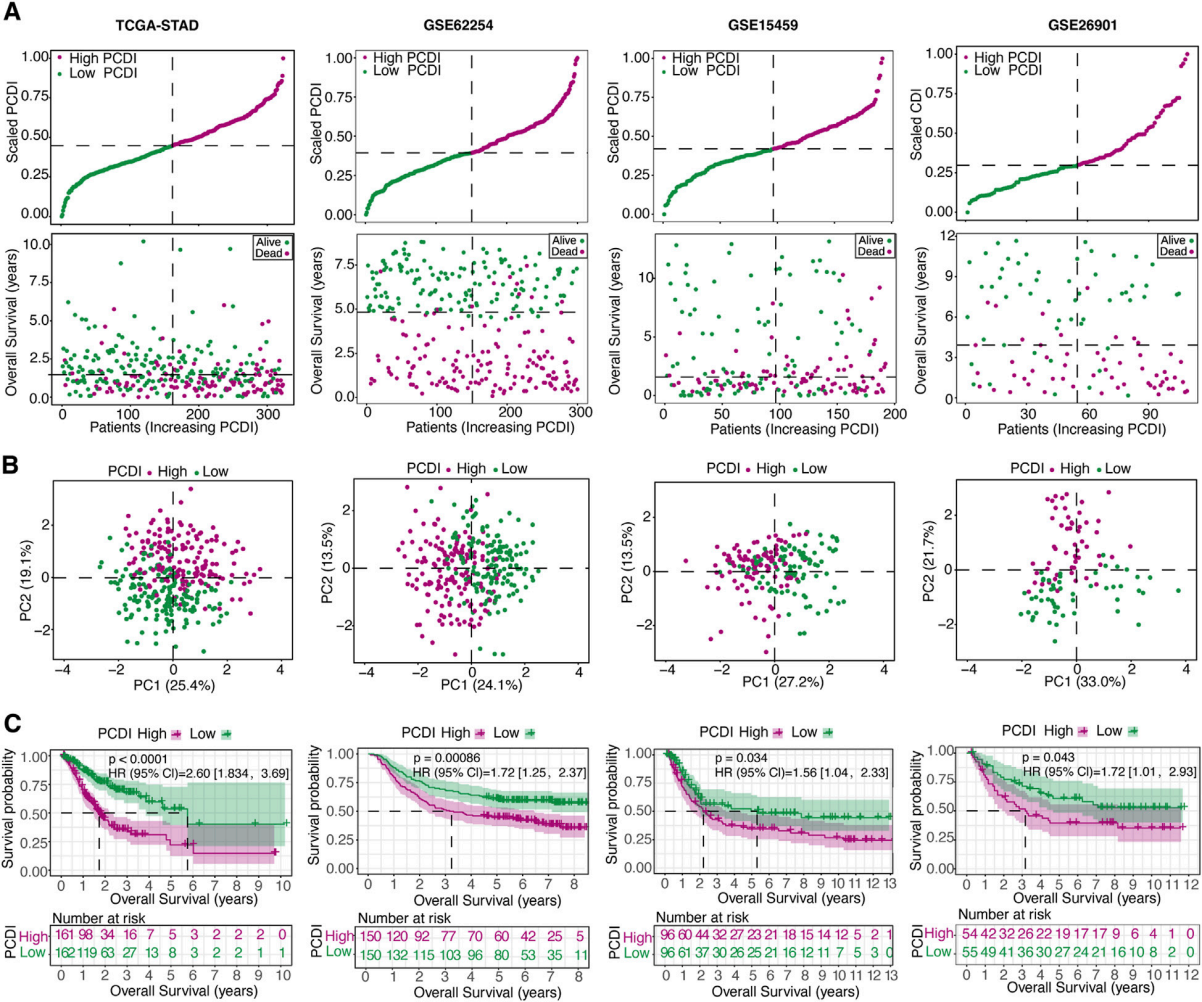
Internal Training and External Validation of the Gene Signature Prediction Model **(A)** Distribution of the scaled PCDI stratified by survival status and overall survival across the TCGA-STAD, GSE62254, GSE15459, and GSE26901 cohorts. **(B)** PCA plots demonstrating PCDI stratification within each cohort. **(C)** Kaplan-Meier survival analyses comparing OS in low- and high-PCDI groups across the cohorts. PCDI, programmed cell death index. PCA, Principal component analysis. OS, overall survival.

We assessed the OS between different PCDI groups within the TCGA-STAD cohort and found that patients with high-PCDI scores exhibited lower survival rates (Figure 3A). Principal component analysis (PCA) confirmed a satisfactory classification based on PCDI (Figure 3B). A marked difference in OS time was noted between the high- and low-PCDI groups, with the latter showing lower death rates (HR = 2.60, 95% CI: 1.83–3.37, *p* < 0.0001; Figure 3C). The validation cohorts (GSE62254, GSE15459, and GSE26901) were also analyzed, demonstrating that a high PCDI score was predictive of poor survival outcomes (Figure 3A). PCA validated the separation of these groups (Figure 3B), and Kaplan-Meier analysis confirmed the significantly lower OS rates in the high-PCDI group (all *p* < 0.05; Figure 3C).

### 3.5 Unsupervised clustering of PCDI model genes

**FIGURE 4:**
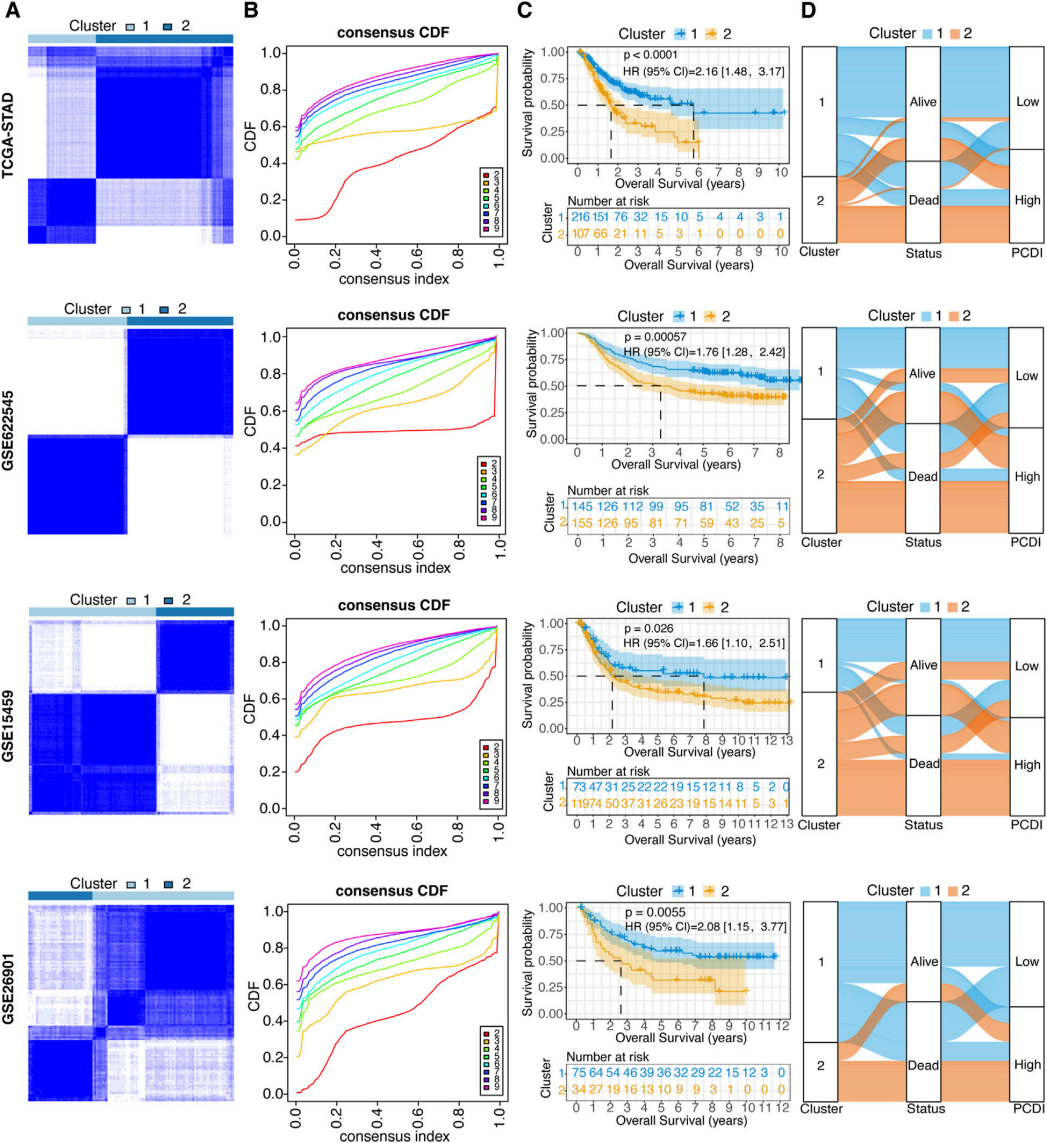
Unsupervised Clustering of PCDI Model Genes **(A)** Stratification of GC patients into two molecular clusters at k = 2, based on the PCDI model gene profile. **(B)** CDF plots illustrating the consensus clustering stability for each k value ranging from 2 to 9. **(C)** Kaplan-Meier survival analysis comparing the prognosis of GC patients across the two identified molecular clusters. **(D)** Alluvial diagram depicting the relationships among molecular clusters, survival status, and PCDI group classification in GC patients. GC, gastric cancer. PCDI, programmed cell death. CDF, cumulative distribution function.

To explore the unidentified subtypes of GC, 12 PCD-related model genes were used to perform a consensus cluster analysis in the TCGA-STAD cohort. We found that when k = 2, the differences among the subgroups were obvious, which indicated that the 323 patients with GC could be well classified into two clusters (Figures 4A, B). An obvious difference was found between the OS time and the two clusters (HR = 2.16, 95% CI: 1.48–3.17, *p* < 0.0001; Figure 4C). Cluster 1 was associated with a favorable prognosis, whereas cluster 2 was associated with a poor prognosis. Similar results were found in the GSE62254 (HR = 1.76, 95% CI: 1.28–2.42, *p* = 0.000057), GSE15459 (HR = 1.66, 95% CI: 1.10–2.51, *p* = 0.026), and GSE26901 cohorts (HR = 2.08, 95% CI: 1.15–3.77, *p* = 0.0055; Figures 4A–C). Moreover, the alluvial diagrams showed that the majority of cluster 1 was associated with a low PCDI, whereas the majority of cluster 2 was associated with a high PCDI (Figure 4D).

### 3.6 Establishment and assessment of the nomogram survival model

**FIGURE 5:**
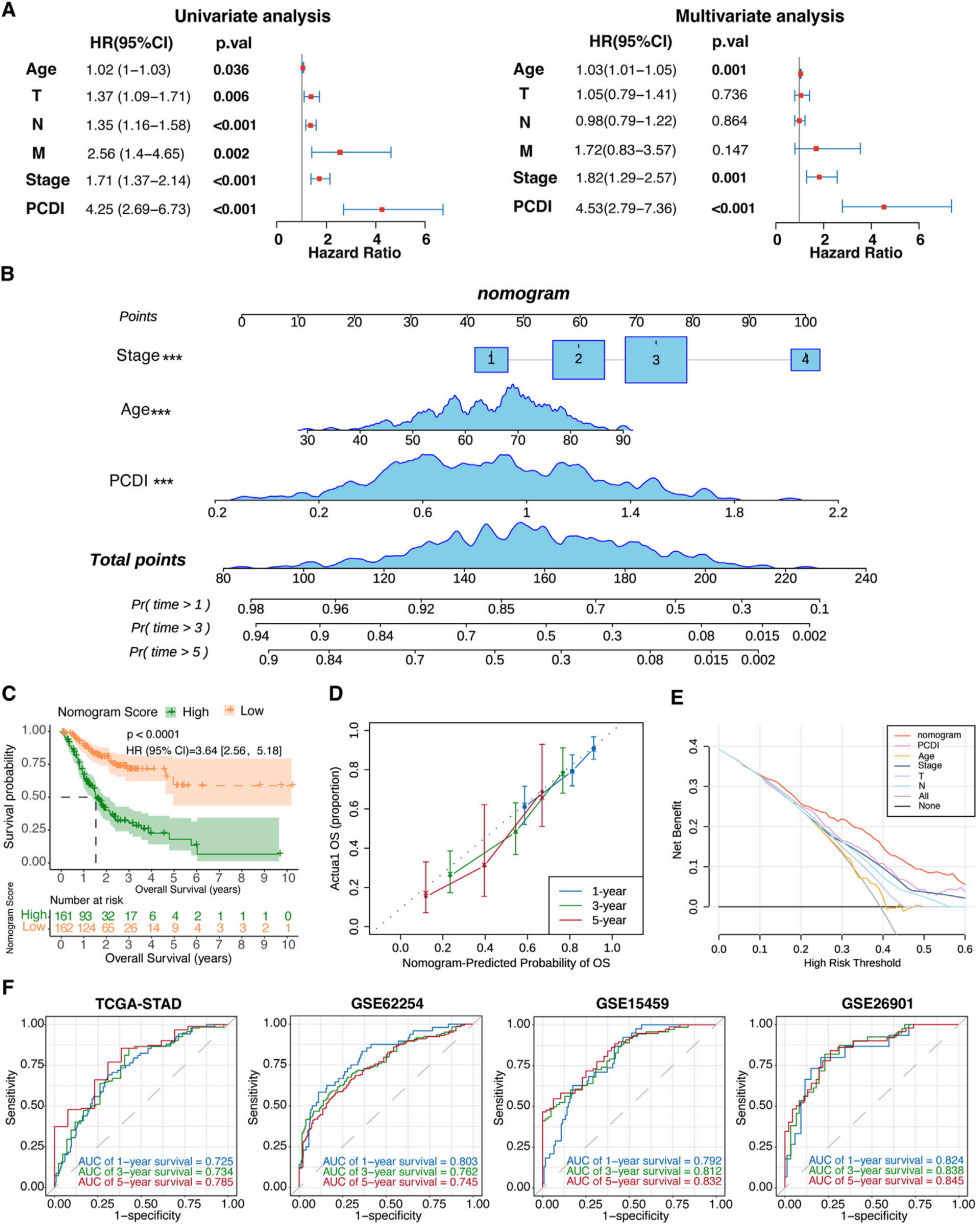
Establishment and Evaluation of the Nomogram Survival Model **(A)** Univariate and multivariate analyses assessing clinicopathologic features and PCDI within the TCGA-STAD cohort. **(B)** Development of a nomogram for predicting 1-year, 3-year, and 5-year OS rates for GC patients. **(C)** Kaplan-Meier survival analyses based on the nomogram scores for the GC patient groups. **(D)** Calibration plots depicting the predicted probabilities for 1-year, 3-year, and 5-year OS within the TCGA-STAD cohort. **(E)** DCA evaluating the nomogram’s utility in predicting 1-year, 3-year, and 5-year OS. **(F)** ROC analysis validating the nomogram’s predictive accuracy across each cohort. PCDI, programmed cell death index. OS, overall survival. GC, gastric cancer. DCA, decision curve analysis. ROC, receiver operating characteristic.

Univariate and multivariate Cox regression analyses were conducted to evaluate the PCDI as an independent prognostic factor. The univariate analysis identified PCDI as a risk factor (HR = 4.25, 95% CI: 2.69–6.73, *p* < 0.001), which was confirmed by multivariate analysis adjusting for confounding factors (HR = 4.53, 95% CI: 2.79–7.36, *p* < 0.001; Figure 5A). A nomogram incorporating the age, stage, and PCDI was established to predict the 1-, 3-, and 5-year OS rates (Figure 5B). A significant survival difference was observed between high- and low-score groups (HR = 3.64, 95% CI: 2.56–5.18, *p* < 0.0001; Figure 5C). Calibration curves demonstrated the accuracy of this model in predicting the OS rates, supported by decision curve analysis (DCA) and area under curve (AUC) assessments, confirming the model’s high predictive accuracy (Figures 5D–F).

### 3.7 Dissection of tumor microenvironment based on PCDI

**FIGURE 6:**
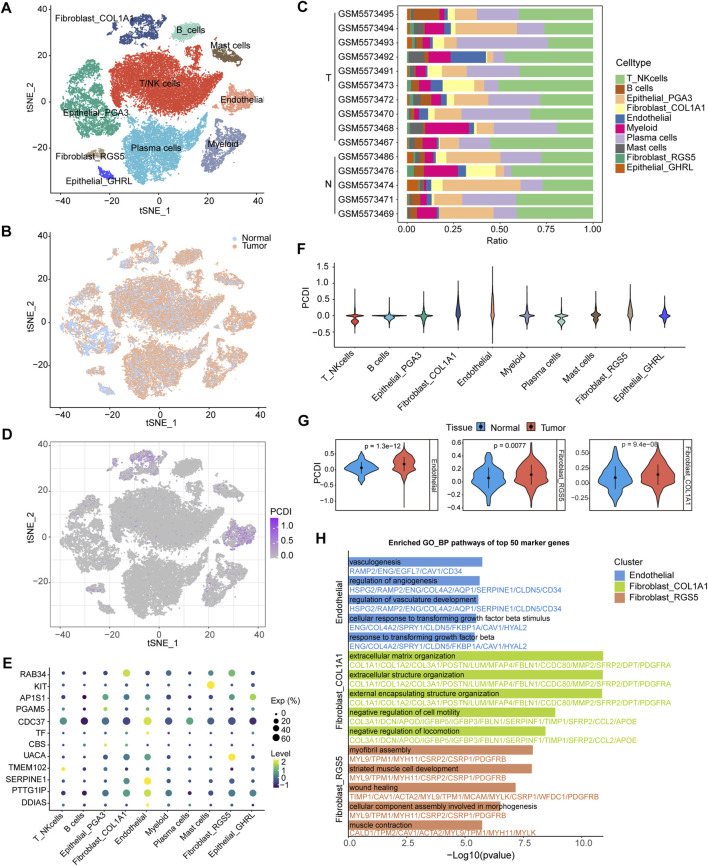
Single-Cell Analysis Based on PCDI **(A)** t-SNE plot visualizing of all cell types from 10 GC tissues and five normal tissues derived from GSE183904. **(B)** t-SNE plot showing the cellular origin as either tumor or normal tissue. **(C)** Bar plots depicting the proportions of cell types of each patient. **(D)** t-SNE plot visualizing the PCDI scores of different cell types. **(E)** Bubble plot illustrating the proportion of cells expressing model genes and the average expression levels of these genes across various cell types. **(F)** Violin plot displaying PCDI scores in each cell type. **(G)** Violin plots comparing the PCDI scores of endothelial, fibroblast_COL1A1 and fibroblast_RGS5 cells between normal and tumor tissues. **(H)** Bar plots showcasing GO_BP pathway enrichment for the top 50 marker genes in endothelial, fibroblast_COL1A1 and fibroblast_RGS5 cells. GC, gastric cancer. T, tumor. N, normal. PCDI, programmed cell death index. GO_BP, biological process pathways of Gene Ontology database.

Further analyses were conducted to elucidate the differences in the TME features between the PCDI groups. We explored PCDI at the single-cell level using data from GSE183904. Major cell types and tissue origins were annotated (Figures 6A, B). The detailed marker genes are shown in Supplementary Figure 5. Bar plots showed the cellular compositions of different samples (Figure 6C). A bubble plot illustrates the proportion of cells expressing the model genes and the average expression levels of these genes across various cell types (Figure 6E). We found that cells with high-PCDI scores were primarily enriched in endothelial cells (ECs), fibroblast_COL1A1, and fibroblast_RGS5 cells (Figures 6D, F). The PCDI scores of these 3 cell types derived from tumor tissues were significantly higher than those derived from normal tissues (Figure 6G). The top 50 marker genes of the ECs were linked to angiogenesis and transforming growth factor beta (TGF-β) related pathways. The top 50 marker genes of the fibroblast_COL1A1 were associated with extracellular matrix (ECM) organization and cell motility regulation pathways. Additionally, the top 50 marker genes of the fibroblast_RGS5 were enriched in the myofibril assembly, wound healing, and muscle-related pathways (Figure 6H; Supplementary Table 3). CIBERSORT algorithms were employed to measure the enrichment scores of immune-related cells in the two PCDI groups, which revealed higher levels of resting CD4^+^ memory T cells and lower levels of activated CD4^+^ memory T cells in the high-PCDI group than in the low-PCDI group, suggesting that the reduction in activated resting CD4^+^ memory T cells may contribute to a poor prognosis in high-PCDI GC patients (Supplementary Figure 6).

### 3.8 Efficacy of PCDI in predicting drug sensitivity

**FIGURE 7:**
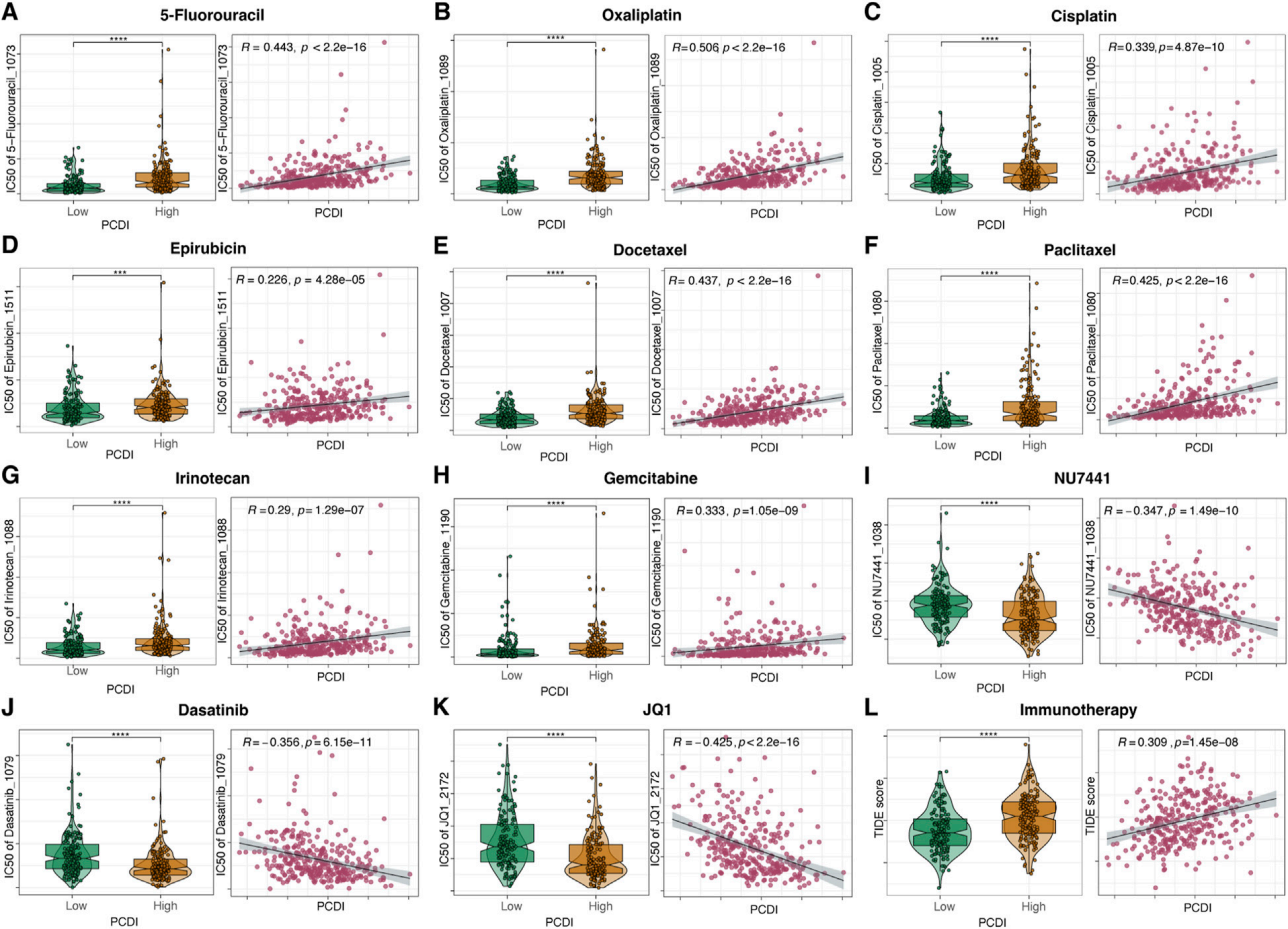
Drug Sensitivity Prediction and Comparison Based on PCDI **(A–K)** Boxplots illustrating the comparison of IC50 values of various drugs between high- and low-PCDI groups, along with the correlation between IC50 and PCDI values in the TCGA-STAD cohort. **(L)** Boxplots showing the comparison of TIDE scores between high- and low-PCDI groups, and the correlation between TIDE scores and PCDI values in the TCGA-STAD cohort. Significance levels are indicated as follows: ns (not significant): *p* > 0.05; *p ≤ 0.05; **p ≤ 0.01; ***p ≤ 0.001; ****p ≤ 0.0001. PCDI, programmed cell death index. IC50, half-maximal inhibitory concentration. TIDE, Tumor Immune Dysfunction and Exclusion.

We assessed the relationship between the PCDI scores and drug sensitivity by calculating the half-maximal inhibitory concentration (IC50) values for various drugs in patients with GC. The correlation landscape between drug sensitivity and PCDI is shown in Figures 7A–K. We found that some commonly used drugs for GC, such as 5-fluorouracil (5-FU), oxaliplatin, cisplatin, epirubicin, docetaxel, paclitaxel, irinotecan, and gemcitabine, had higher IC50 values in the high PCDI group, indicating resistance. In contrast, the IC50 values for NU7441, Dasatinib, and JQ1 were lower in the high-PCDI group, suggesting sensitivity to these drugs. Additionally, the TIDE score assessment revealed that patients with low PCDI were associated with lower TIDE scores, suggesting that these patients may benefit more from immunotherapy (Figure 7L).

## 4 Discussion

In this study, we performed a comprehensive analysis of 14 diverse PCD modalities and established a signature called the PCDI to predict the prognosis of patients with GC within the TCGA-STAD cohort. This signature was further validated with excellent performance across three external cohorts. A nomogram integrating clinical characteristics with the PCDI was constructed, demonstrating robust predictive capabilities. Moreover, our findings highlight a significant correlation between the PCDI, the TME, and drug sensitivity in GC, all of these underscoring the potential clinical utility of the PCDI in guiding personalized treatment decisions.

Functional enrichment analysis of PCD-related DEGs revealed significant enrichment of cell death pathways such as apoptosis, autophagy, and necroptosis, further validating the robustness of our model construction. This underscores the close association between various PCD patterns and the development and metastasis of tumors. Consequently, we identified PCD-related genes that significantly affected the survival of patients with GC for subsequent analyses. We developed and validated a prognostic signature comprising 12 PCD-related genes (CBS, SERPINE1, RAB34, KIT, TF, AP1S1, PGAM5, TMEM102, CDC37, DDIAS, PTTG1IP, and UACA) in patients with GC. CBS is a crucial enzyme in the methionine metabolism pathway. It was reported that high CBS levels were associated with a poor OS rate in GC patients receiving adjuvant chemotherapy (Zhao et al., 2021). Our study revealed that the CBS expression was lower in GC samples, and the relatively lower expression was associated with a better prognosis. SERPINE1, known for its role in angiogenesis and metastasis, is highly expressed and significantly associated with a poor prognosis in GC, corroborating our findings (Li et al., 2019). RAB34, which regulates cell morphology and motility, showed no significant differences in expression between GC and normal tissues; however, higher levels of RAB34 in GC tissues were associated with a worse prognosis, consistent with findings in other cancers, such as breast cancer, hepatocellular carcinoma, and glioma (Sun et al., 2018a; Wu et al., 2017; Hou et al., 2022). KIT, a classical proto-oncogene that encodes a receptor tyrosine kinase responsive to stem cell factor, has been found to promote tumor development and progression in various cancers through overexpression or mutations (Sheikh et al., 2022). Our findings suggested that higher KIT expression levels in GC tissues were associated with a poorer prognosis. TF, which is a type of iron-binding protein involved in iron transport, was found to be highly expressed in GC, and its high expression was linked to poor prognosis in our study (Torti and Torti, 2013). AP1S1, a part of the adaptor protein complex involved in clathrin-mediated endocytosis, is highly expressed in breast cancer and glioblastoma and is associated with poor prognosis (Zheng et al., 2022; Wu et al., 2022). In contrast, our research indicated that although AP1S1 was overexpressed in GC tissues compared to normal tissues, it served as a protective factor in our model. PGAM5, an atypical mitochondrial serine/threonine phosphatase, promotes GC cells proliferation by positively regulating the PI3K/AKT signaling pathway (Meng et al., 2023). Our study observed significantly elevated PGAM5 levels in GC tissues; however, patients with higher PGAM5 levels had a better prognosis. TMEM102 is a transmembrane protein that has been identified as a proapoptotic molecule involved in GM-CSF deprivation-induced apoptosis (Kao et al., 2008). Overexpression of TMEM102 implicates poor prognosis and chemoresistance in patients with epithelial ovarian carcinoma (Tai et al., 2022). TMEM102 was significantly upregulated in GC tissues compared to normal tissues, suggesting its potential as a prognostic factor for GC. CDC37 is a key determinant of client kinase recruitment to the HSP90 chaperoning system. Patients with CDC37-high metastatic colorectal cancer benefited more from anti-VEGF therapy (Arai et al., 2024). CDC37 serves as a protective factor against GC, which was confirmed in our study (Wang et al., 2023). DDIAS stimulates cancer cell proliferation and cell cycle progression by inhibiting DNA damage-induced apoptosis (Im et al., 2023). Although significantly higher expression levels of DDIAS were observed in GC tissues than in normal tissues, DDAIS served as a protective factor in the model. PTTG1IP is an oncogenic protein that participates in the metaphase-anaphase transition of the cell cycle through the activation of securin (PTTG1) (Repo et al., 2017). The overexpression of PTTG1IP was associated with poor prognosis in epithelial ovarian cancer, thyroid cancer and head and neck squamous cell carcinoma (Ma et al., 2023; Read et al., 2018; Read et al., 2017). Our study revealed similar results. Higher UACA expression in normal cells results in lower extracellular Par-4 levels, leading to reduced tumor apoptosis and worse prognosis (Burikhanov et al., 2013). UACA is upregulated in hepatocellular carcinoma and promotes cell proliferation and invasion (Sun et al., 2018b). Additionally, its elevated expression is associated with poor OS in breast cancer (Zhu et al., 2022). Similar trends were observed in GC, indicating worse prognosis with higher UACA expression.

To explore the potential relationships between the PCDI and other clinical characteristics, we conducted subgroup analyses on PCDI. The results revealed that a high PCDI score was significantly associated with mortality outcomes. No significant differences were noted in the PCDI across subgroups based on sex, the AJCC Stage, N stage, or M stage, indicating PCDI’s robust independence as a novel clinical feature for GC patients. Additionally, we observed that PCDI levels were significantly lower in patients with T1 tumors than in those with T2-4 tumors. Intriguingly, we detected variations in PCDI across TCGA molecular subtypes, with patients with GS and CIN patients generally exhibiting higher PCDIs, whereas patients with EBV and MSI patients presented with lower PCDIs. Research has shown that MSI and EBV subtypes generally have better prognoses, while GS subtypes have the worst prognoses (Sohn et al., 2017). Furthermore, EBV and MSI subtypes are likely to benefit from immunotherapy, whereas the CIN and GS subtypes are less responsive to such treatments (Alsina et al., 2023). Our findings are consistent with these observations. Additionally, among different MSI statuses, patients with MSI-H had lower PCDI than in MSI-L and MSS patients, and had a better prognosis, which was consistent with the TCGA molecular subtype. Overall, these findings underscore that the PCDI is a relatively independent prognostic marker. The independent prognostic value of PCDI was further validated using both univariate and multivariate Cox regression analyses. Moreover, we developed a nomogram model that incorporated the PCDI with relevant clinical parameters. The effectiveness of this model was validated, and its clinical utility was demonstrated. The prognostic nomogram model exhibited strong predictive capabilities for 1-, 3-, and 5-year OS, highlighting its potential for enhancing patient management and outcome prediction in clinical settings.

Single-cell analysis revealed that the cell types with high PCDI scores mainly included ECs and two clusters of fibroblast cells. Furthermore, the PCDI scores of these cells from tumor tissues were significantly higher than those from normal tissues. The marker genes of ECs were primarily enriched in angiogenesis and TGF-β related pathways. Angiogenesis is essential for tumor progression because tumor-endothelial interactions promote malignant vascularization and influence the proliferative and metastatic potential of cancer cells (Yao and Zeng, 2023). TGF-β stimulates the secretion of factors like VEGF-A, which further promotes angiogenesis. The newly formed vessels provide nutrients for cancer growth and dissemination (Derynck et al., 2021). In addition, TGF-β signaling plays a crucial role in cancer resistance to chemotherapy, targeted therapy, and immunotherapy (Zhang et al., 2021). We speculated that some of the identified ECs were tumor-associated ECs (TAECs). The marker genes of fibroblast_COL1A1 were enriched in the pathways related to ECM formation. Cancer-associated fibroblasts (CAFs) can remodel the ECM by secreting large amounts of collagen and fibronectin, creating barriers that hinder the penetration of drugs and immune cells into tumors, thereby reducing treatment efficacy (Biffi and Tuveson, 2021). The marker genes of fibroblast_RGS5 were associated with myofibril assembly and wound healing, suggesting that these cells might linked to myofibroblastic CAFs (myoCAFs) (Mellone et al., 2022). MyoCAF-rich stroma is linked to a poor prognosis and contributes to several hallmarks of malignancy (Mellone et al., 2022). Moreover, myoCAF-rich cancers have low levels of infiltrating T cells and promote resistance to various immunotherapies (Mellone et al., 2022). Collectively, we hypothesized that PCDI model genes may be involved in the transition of ECs to TAECs and fibroblasts to CAFs, which plays a significant role in tumor onset and progression, and ultimately leads to adverse prognoses. In addition, analysis of immune cell infiltration indicated that a decrease in the transition of CD4^+^ memory T cells from a resting state to an activated state might contribute to the poor prognosis of GC patients with a high PCDI.

Using drug sensitivity prediction, we discovered that patients with a high PCDI were resistant to traditional chemotherapeutic drugs, such as 5-FU, oxaliplatin, cisplatin, epirubicin, docetaxel, and paclitaxel, which may explain their poor prognosis. However, we were surprised to identify NU7441, Dasatinib, and JQ1 as potential therapeutic drugs for patients with a high PCDI score. NU7441, a DNA-PKcs inhibitor, has been found to increase the sensitivity of radioresistant GC cells to radiotherapy through the cleaved-caspase3/γH2AX signaling pathway (Geng et al., 2019). Dasatinib, a multi-target kinase inhibitor that targets BCR-ABL, SRC family kinases, and various cancer kinases, has been identified as an efficient inhibitor of GC proliferation (Montenegro et al., 2020). Additionally, Dasatinib synergizes with platinum-based drugs to combat GC (Wang et al., 2022). JQ1, a BRD4 inhibitor, potently inhibited of the cell growth and malignant progression of GC by downregulating chromatin accessibility and inactivating the RUNX2/NID1 signaling pathway (Zhou et al., 2020). Moreover, the application of BRD4 inhibitors can enhance the anti-tumor effects of Dasatinib in GC (Shen et al., 2022). Although the results indicate that GC patients with a high PCDI may benefit less from immunotherapy, the aforementioned targeted therapies could achieve promising therapeutic effects.

In this study, we developed a predictive model based on PCD-related genes to offer insights into the prognostic potential of PCD in GC. However, there are several limitations. Firstly, the model was developed using retrospective data, which may have introduced biases affecting the generalizability of our findings. Future prospective studies and validations in independent cohorts are essential to confirm the clinical utility of the PCDI. Secondly, the functional roles of specific model genes in the context of GC may not have been explored. Although our analysis identified certain genes as potential biomarkers, further investigation is required to understand the molecular mechanisms by which these genes affect GC progression and patient outcomes. Expression should be confirmed not only at the RNA level, but also at the protein level and in terms of functional activity. Thirdly, the decision-making utility of our predictive model has not yet been tested in clinical trials. Its effectiveness in guiding treatment decisions, particularly for targeted therapy in GC patients with a high PCDI, requires validation through adequately powered, multicenter, phase 3 randomized controlled trials. Further studies are needed to elucidate the role of these genes in GC and confirm the predictive accuracy of this model in clinical applications.

## 5 Conclusion

In conclusion, we identified 12 PCD-related genes that were strongly associated with GC prognosis. Additionally, we proposed a novel signature, the PCDI, which demonstrated robust performance for predicting the survival and therapeutic response, and showed potential utility in assisting clinicians in providing more efficient and personalized treatments for patients with GC.

## Data Availability

Publicly available datasets were analyzed in this study. This data can be found here: TCGA: https://www.cancer.gov/ccg/research/genome-sequencing/tcga GEO: https://www.ncbi.nlm.nih.gov/geo/.
