# Supplementary material for: Development and validation of a programmed cell death index to predict the prognosis and drug sensitivity of gastric cancer
Source: Front Pharmacol. 2024 Dec 18;15:1477363. doi: 10.3389/fphar.2024.1477363 (PMC11688280; doi:10.3389/fphar.2024.1477363)
Supplement: Supplementary file 2 [file DataSheet1.docx]

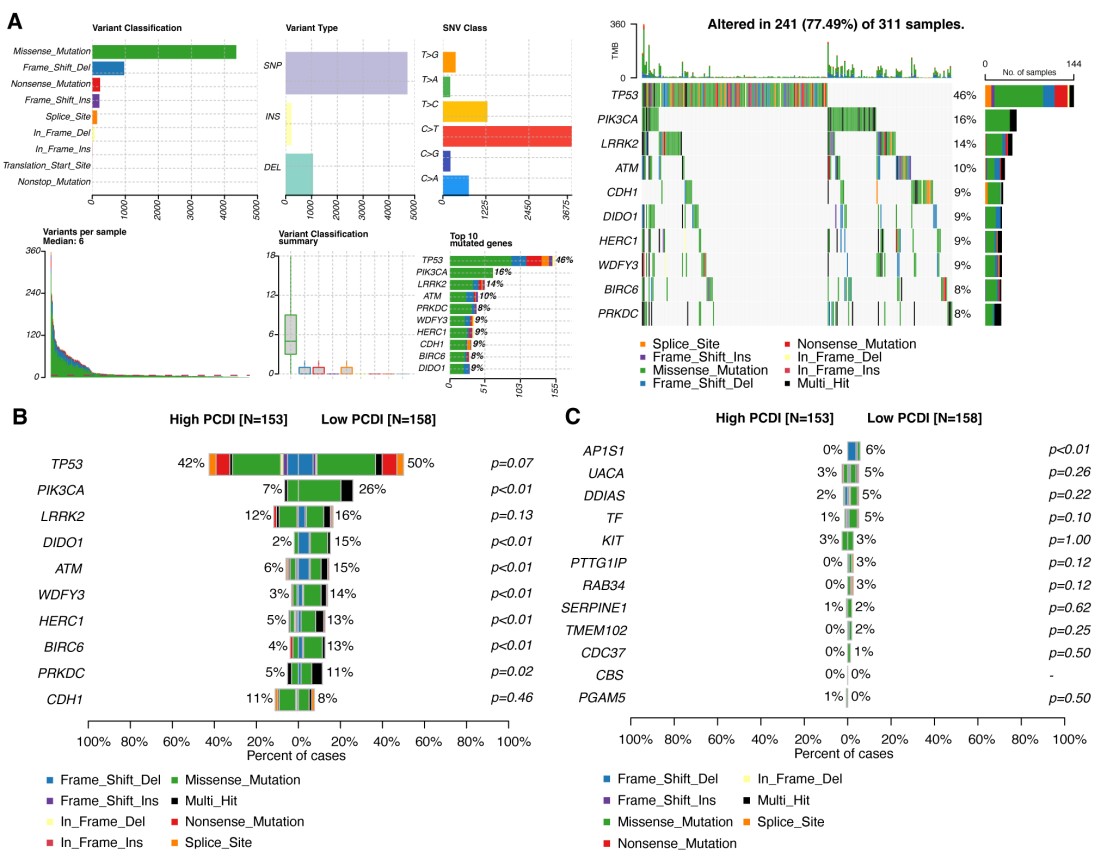


**Supplementary Figure 1.** (A) Landscape of masked somatic mutations in PCD-related genes in the TCGA-STAD cohort. (B) Comparison of the top 10 mutated PCD-related genes between high and low PCDI groups in the TCGA-STAD cohort. (C) Comparison of mutations in model genes between high and low PCDI groups in the TCGA-STAD cohort. PCD, programmed cell death. PCDI, programmed cell death index.


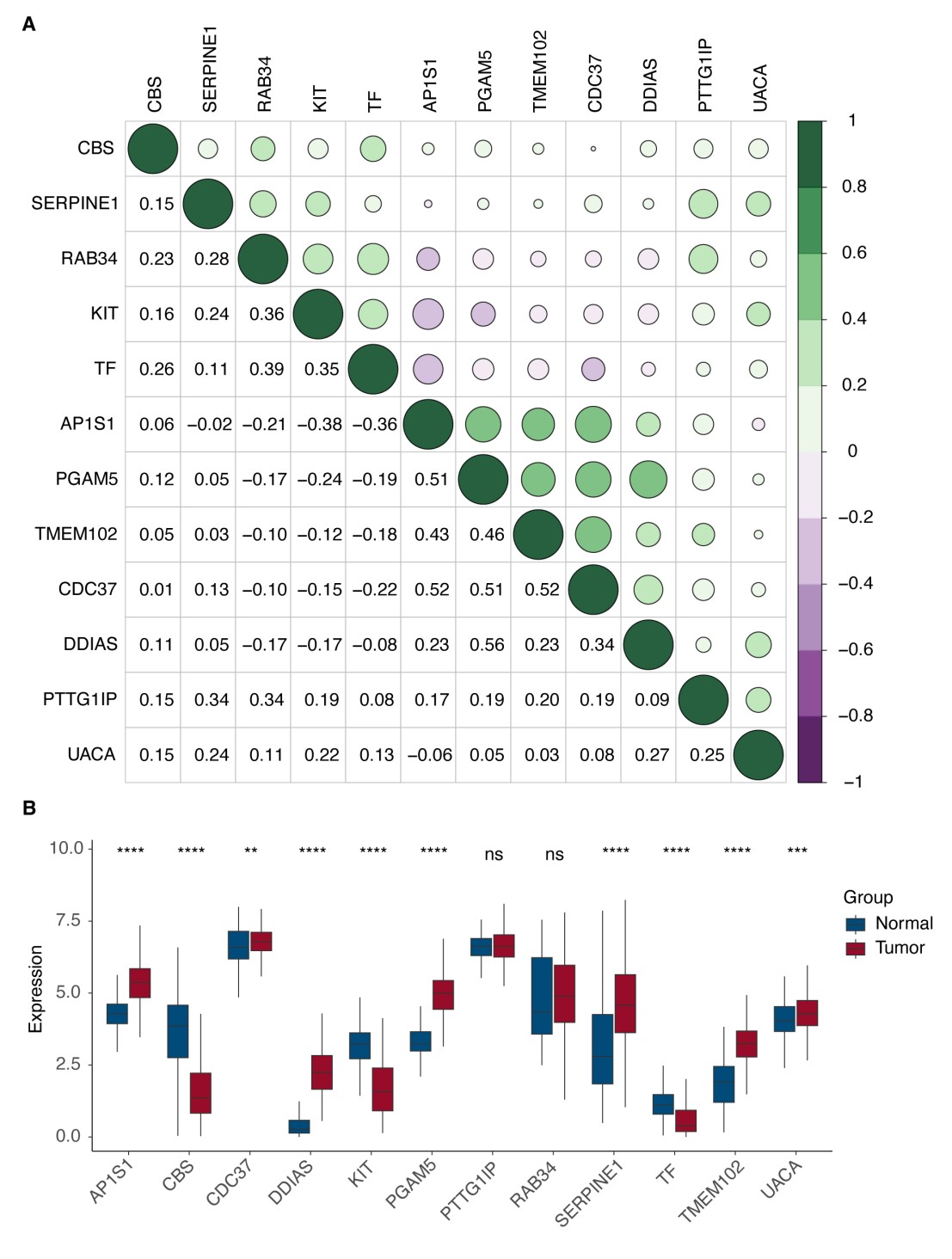


**Supplementary Figure 2.** (A) A correlogram displaying Spearman’s correlation coefficients (r) for twelve model genes based on the TCGA-STAD cohort, with sector areas proportional to the r coefficient. (B) Bar plot showing the differential expression levels of PCDI genes between tumor and normal samples in the TCGA-STAD cohort. Significance levels are indicated as follows: ns (not significant): p > 0.05; *: p ≤ 0.05; **: p ≤ 0.01; ***: p ≤ 0.001; ****: p ≤ 0.0001.


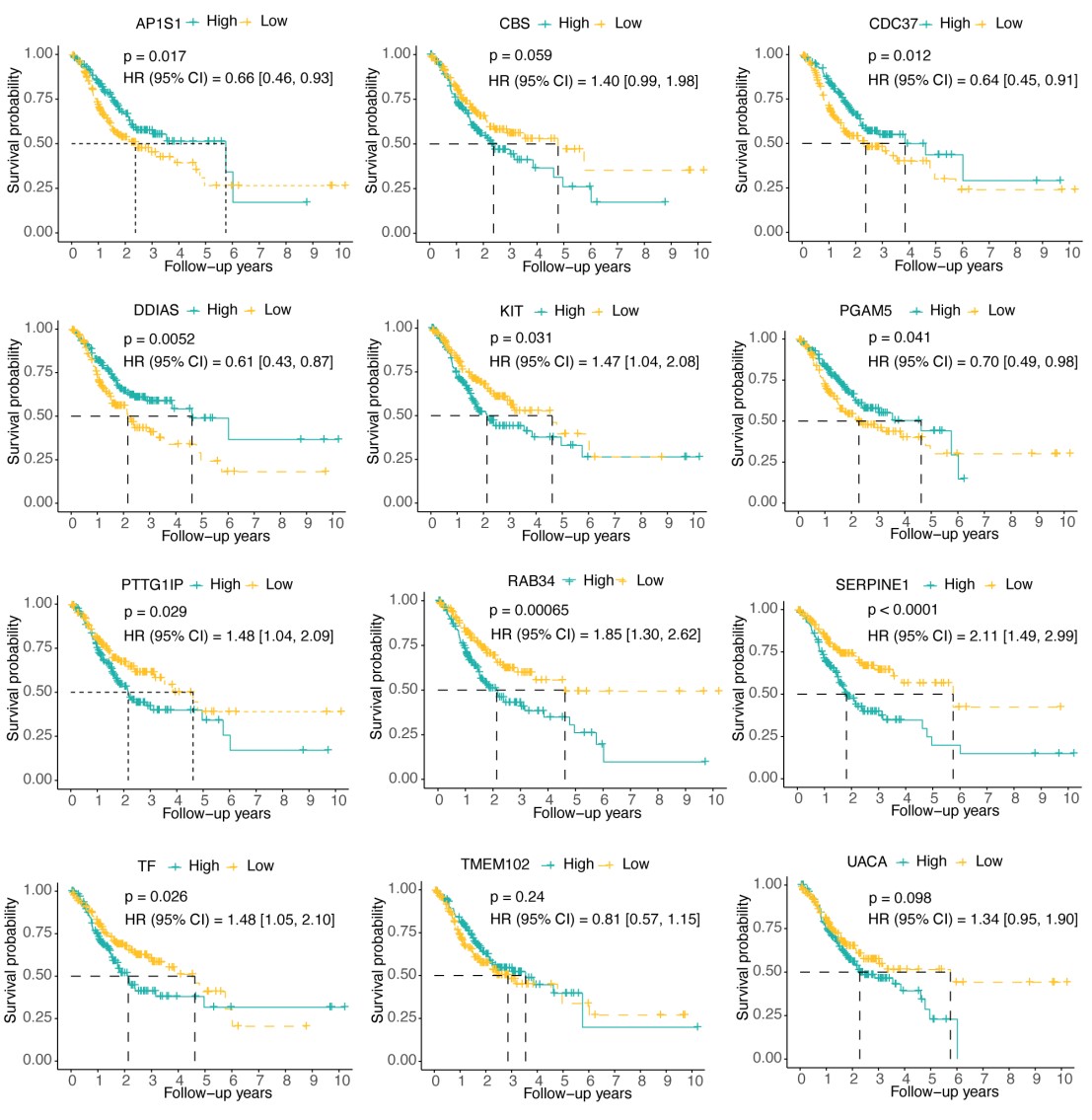


**Supplementary Figure 3.** Kaplan-Meier survival analysis of each model genes in the TCGA-STAD cohort, with samples divided into high- and low-expression groups using the median expression

(green: high-expression group; yellow: low-expression group).


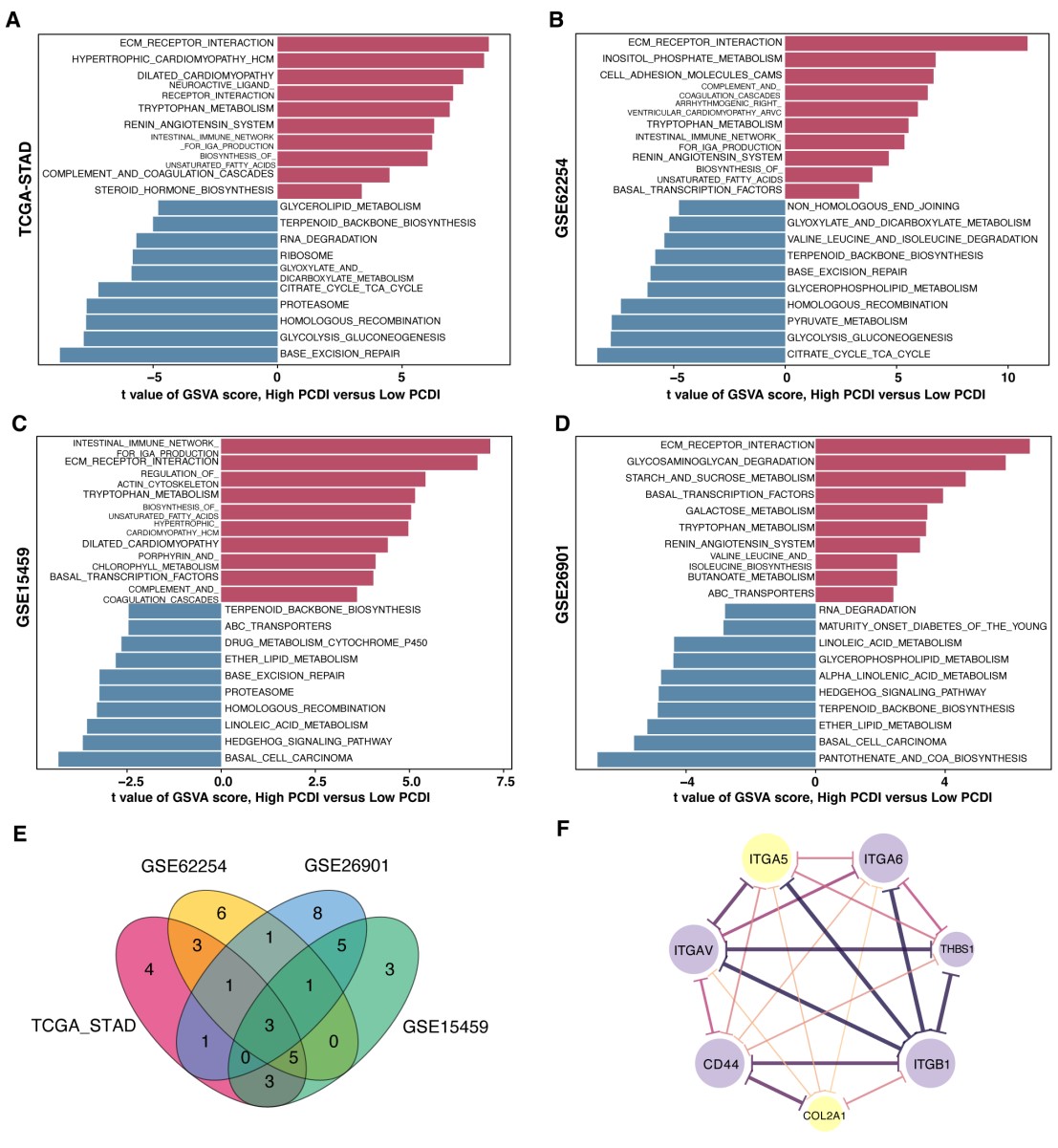


**Supplementary Figure 4.** (A-D) Bar plots displaying the top 20 upregulated and downregulated pathways identified through GSVA across four cohorts. (E) Venn diagram showing two commonly upregulated KEGG pathways ("ECM RECEPTOR INTERACTION", "TRYPTOPHAN METABOLISM") and one commonly downregulated pathway ("TERPENOID BACKBONE BIOSYNTHESIS") across the cohorts. (F) PPI network analysis of seven PCD-related genes involved in the three pathways mentioned (purple: upregulated genes, yellow: downregulated genes, the thickness and color depth of lines revealed the strength of correlations). PCD, programmed cell death. PCDI, programmed cell death index. GSVA, gene set variation analysis. KEGG, Kyoto Encyclopedia of Genes and Genomes. PPI, protein-protein interaction.


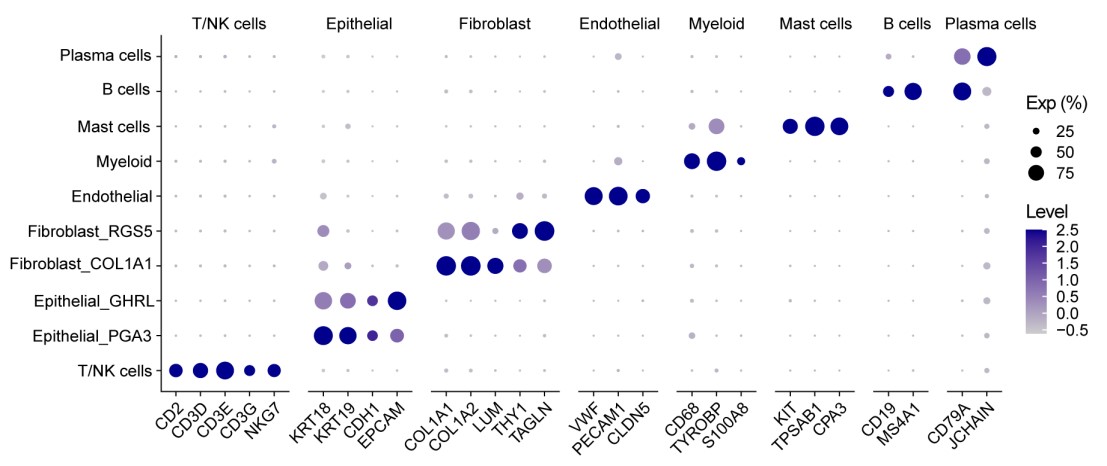


**Supplementary Figure 5.** Bubble plot illustrating the proportion of cells expressing marker genes and the average expression levels of these genes across various cell types.


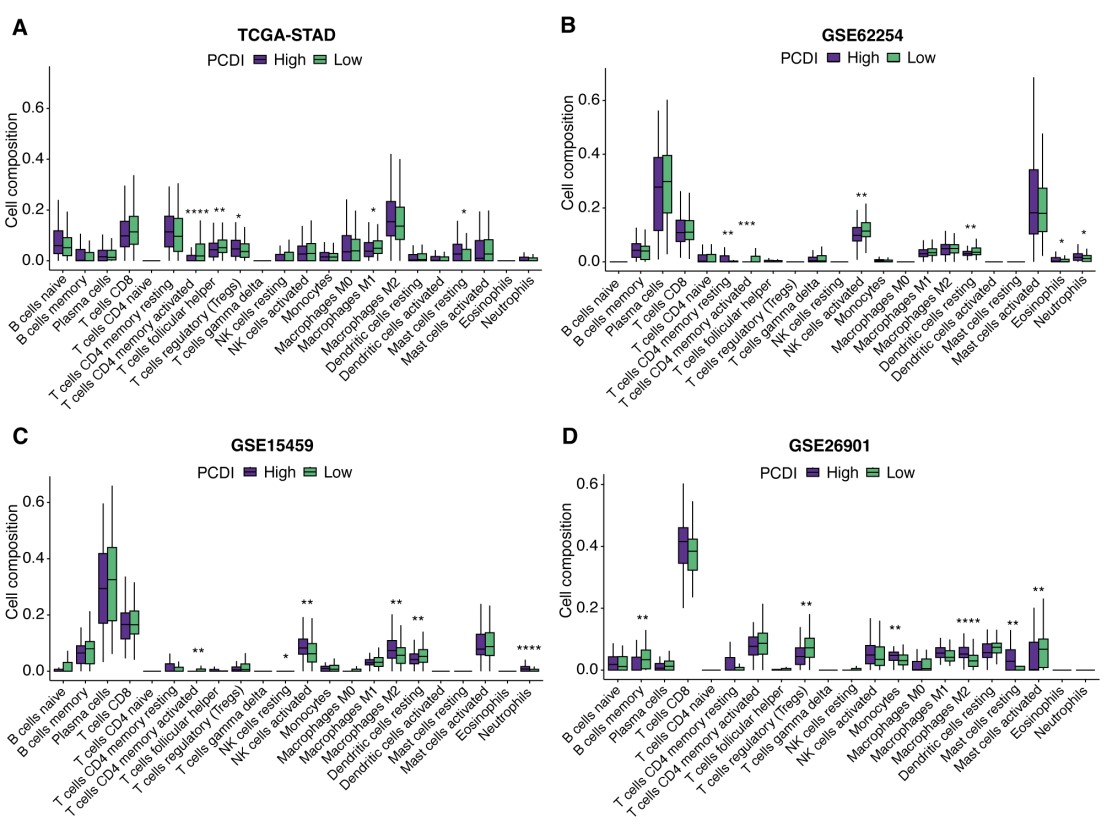


**Supplementary Figure 6.** (A-D) Bar plots comparing immune cell infiltration between high and low PCDI groups within the TCGA-STAD, GSE62254, GSE15459, and GSE26901 cohorts using the CIBERSORT analysis. PCDI, programmed cell death index.
